# Single-cell sequencing analysis of peripheral blood in patients with moyamoya disease

**DOI:** 10.1186/s13023-023-02781-8

**Published:** 2023-07-03

**Authors:** Qikai Tang, Wenjun Li, Jie Huang, Yuting Wu, Chenfeng Ma, Yiming Tu, Qianmiao Zhu, Jiacheng Lu, Jiaheng Xie, Yu Liu, Xiaoman Mao, Wei Wu

**Affiliations:** 1 Department of Neurosurgery, The First Affiliated Hospital of Nanjing Medical University, Jiangsu Province Hospital, Nanjing, 210029 Jiangsu China; 2 State Key Laboratory of Reproductive Medicine, Nanjing Medical University, Nanjing, China; 3 Department of pharmacy, university of Southern California, Los Angeles, CA USA; 4 Department of Neurosurgery, Zhongda Hospital, Southeast University, Nanjing, 210009 Jiangsu P.R. China; 5 Department of Burn and Plastic Surgery, Jiangsu Province Hospital, The First Affiliated Hospital of Nanjing Medical University, Nanjing, 210029 Jiangsu China

**Keywords:** Moyamoya disease, Single-cell sequencing, Weighted co-expression network analysis(WGCNA), B cell, Pathways

## Abstract

**Background:**

At present, the etiology of moyamoya disease is not clear, and it is necessary to explore the mechanism of its occurrence and development. Although some bulk sequencing data have previously revealed transcriptomic changes in Moyamoya disease, single-cell sequencing data has been lacking.

**Methods:**

Two DSA(Digital Subtraction Angiography)-diagnosed patients with moyamoya disease were recruited between January 2021 and December 2021. Their peripheral blood samples were single-cell sequenced. CellRanger(10 x Genomics, version 3.0.1) was used to process the raw data, demultiplex cellular barcodes, map reads to the transcriptome, and dowm-sample reads(as required to generate normalized aggregate data across samples). There were 4 normal control samples, including two normal samples GSM5160432 and GSM5160434 of GSE168732, and two normal samples of GSE155698, namely GSM4710726 and GSM4710727. Weighted co-expression network analysis was used to explore the gene sets associated with moyamoya disease. GO analysis and KEGG analysis were used to explore gene enrichment pathways. Pseudo-time series analysis and cell interaction analysis were used to explore cell differentiation and cell interaction.

**Results:**

For the first time, we present a peripheral blood single cell sequencing landscape of Moyamoya disease, revealing cellular heterogeneity and gene expression heterogeneity. In addition, by combining with WGCNA analysis in public database and taking intersection, the key genes in moyamoya disease were obtained. namely PTP4A1, SPINT2, CSTB, PLA2G16, GPX1, HN1, LGALS3BP, IFI6, NDRG1, GOLGA2, LGALS3. Moreover, pseudo-time series analysis and cell interaction analysis revealed the differentiation of immune cells and the relationship between immune cells in Moyamoya disease.

**Conclusions:**

Our study can provide information for the diagnosis and treatment of moyamoya disease.

## Introduction

Moyamoya disease is a cerebrovascular disease characterized by chronic progressive stenosis or occlusion at the end of bilateral internal carotid artery and the beginning of the anterior cerebral artery and middle cerebral artery, with secondary abnormal vascular network formation at the skull base [1]. Moyamoya disease was first proposed and named by Japanese scholars in 1957 [2]. “Moyamoya,“ which means “smoke” in Japanese, graphically describes the patient’s network of compensatory abnormal blood vessels swirling like smoke [3, 4]. In recent years, both the incidence and prevalence of moyamoya disease have gradually increased, which may depend on advances in diagnostic techniques [5, 6]. Globally, people in Asia have a higher incidence of moyamoya disease, especially in Japan and South Korea [7]. Notably, two peaks were found in the age of onset of Moyamoya disease: 5–10 years and 25–49 years [8–10]. The main clinical manifestation of Moyamoya disease is ischemic or hemorrhagic stroke, which has a high mortality rate [11]. However, the pathogenesis of moyamoya disease is still unknown. Previous genomics studies have identified the RNF213 gene as a susceptibility gene in moyamoya disease [12, 13]. However, cellular heterogeneity, transcriptome changes, signaling pathway changes, and intercellular communication in Moyamoya disease remain unclear. In-depth exploration is urgently needed to provide reference for the pathogenesis and treatment of moyamoya disease.

At present, single cell sequencing technology is a revolution in life science research [14]. Although the rapid development of next generation sequencing (NGS) technology and the third generation sequencing (TGS) technology has caused a huge change in the field of biological research, but the sequencing results are a representation of the cells as a whole [15, 16]. However, due to cell heterogeneity, the genetic information of cells with the same phenotype may be significantly different, and much information with low abundance will be lost in the overall characterization [17]. In order to make up for the limitations of traditional high-throughput sequencing, single-cell sequencing technology emerged. It performs high-throughput sequencing of the genome, transcriptome, and epigenome at the single cell level. It can reveal the gene structure and gene expression state of a single cell and reflect the heterogeneity between cells. It plays an important role in the fields of tumor, developmental biology, microbiology, neuroscience and so on, and is becoming the focus of life science research [18–20]. However, there is still a blank in the field of sequencing peripheral blood single cells in human patients with Moyamoya disease, which makes it difficult for us to deeply understand the transcriptome expression of various cells and the immune function in the microenvironment of moyamoya disease.

To explore the pathogenesis of moyamoya disease, single cell sequencing was performed in patients with moyamoya disease and normal controls. We performed weighted co-expression network analysis, enrichment analysis, protein-protein interaction analysis, pseudo-time series analysis, and intercellular communication analysis, in order to provide a reference for diagnosis and treatment of moyamoya disease and identification of markers.

## Methods

### Single cell data acquisition and processing

Two DSA(Digital Subtraction Angiography)-diagnosed patients with moyamoya disease were recruited between January 2021 and December 2021. They signed informed consent and peripheral blood samples were used for single-cell sequencing. The Ethics Committee of the First Affiliated Hospital of Nanjing Medical University approved the study (No. 2021-SR-165). CellRanger(10× Genomics, version 3.0.1) was used to process the raw data, demultiplex cellular barcodes, map reads to the transcriptome, and dowm-sample reads(as required to generate normalized aggregate data across samples). There were 4 normal control samples, including two normal samples GSM5160432 and GSM5160434 of GSE168732, and two normal samples of GSE155698, namely GSM4710726 and GSM4710727.

### Quality control of single cell sequencing data

The “Seurat” package was used to process and analyze single cell sequencing data [21]. The criteria for inclusion of genes for subsequent analysis are as follows: (1) Genes expressed in at least 3 cells. (2) Genes whose expression level is above 200. The inclusion criteria were as follows: (1) The gene expression level was greater than 200 but less than 4000. (2) The percentage of mitochondrial gene is less than 25. (3) The total gene expression is less than 15,000.

### Processing of data

First, the “LogNormalize” method is used to standardize data. Subsequently, the number of highly variable genes was set to 3000, and the method was “vst” to search for highly variable genes. The SCTransform function of the “Seurat” package was used to integrate the data and eliminate interference from the cell cycle and mitochondria. Set dims to 20 and use the tSNE method to reduce the dimensions. The “KNN” method was used to cluster cells, and K.Paam was set as 20, dims = 20, resolution = 0.8, random.seed = 2021. The “SingleR” package was used to annotate the cells.

### Weighted gene co-expression Network Analysis (WGCNA)

The “sample” function was used to randomly select 2000 cells, including both Moyamoya disease and normal samples, for subsequent analysis. In this study, genes with variance fluctuation in the top 50% were selected for subsequent analysis. If the soft threshold ranges from 1 to 10, the step is (1) From 12 to 20, the step size is (2) The “WGNCA” package pickSoftThresgold function is used to explore the soft threshold changes. Set deepSplit = 2, the minimum number of module genes was 100, and the genes were clustered into different modules. Finally, each module was associated with the phenotype to find the module gene most associated with moyamoya disease.

### Gene Ontology(GO) enrichment analysis

Gene Ontology(GO) is a database established by the Gene Ontology Consortium, which aims to establish a database applicable to various species to define and describe the functions of genes and proteins [22]. GO enrichment analysis is divided into three categories, namely Biological Process (BP), Cellular Component (CC) and Molecular Function (MF). We used GO enrichment analysis to explore the enrichment function of gene sets.

### Kyoto Encyclopedia of genes and genomes (KEGG) enrichment analysis

KEGG(Kyoto Encyclopedia of Genes and Genomes) database is a database that systematically analyzes gene functions, genomic information and functional information, including metabolic pathways database, hierarchical classification database, gene database, genome database, etc [23]. By comparing the studied genes with the pathway gene set in the database, the enriched pathways were obtained.

### Pseudo-time series analysis and cell interaction analysis

The “monocle2” package was used for cell differentiation trajectory analysis. Genes with average expression greater than 0.1 were selected for subsequent analysis. Set max_components = 2 and method = DDRTree to reduce the sample dimensions. Cell differentiation was shown by tSNE diagram. The “CellChat” packet was used for cell interaction analysis. The database “Secreted Signaling” was selected and the minimum number of cells was set to 10.

### Statistical analysis

The method used to search for genes associated with moyamoya disease is WGCNA. The Chi-squared test and Fisher’s exact test were performed to compare the clinical variables. The “Seurat” package was used to process and analyze single cell sequencing data. The ClusterProfile package was used for enrichment analysis. The “monocle2” package was used for pseudo temporal analysis. The software used was R software, version 4.0.5. Without specific description, p < 0.05 was considered statistically significant.

## Results

**Fig. 1:**
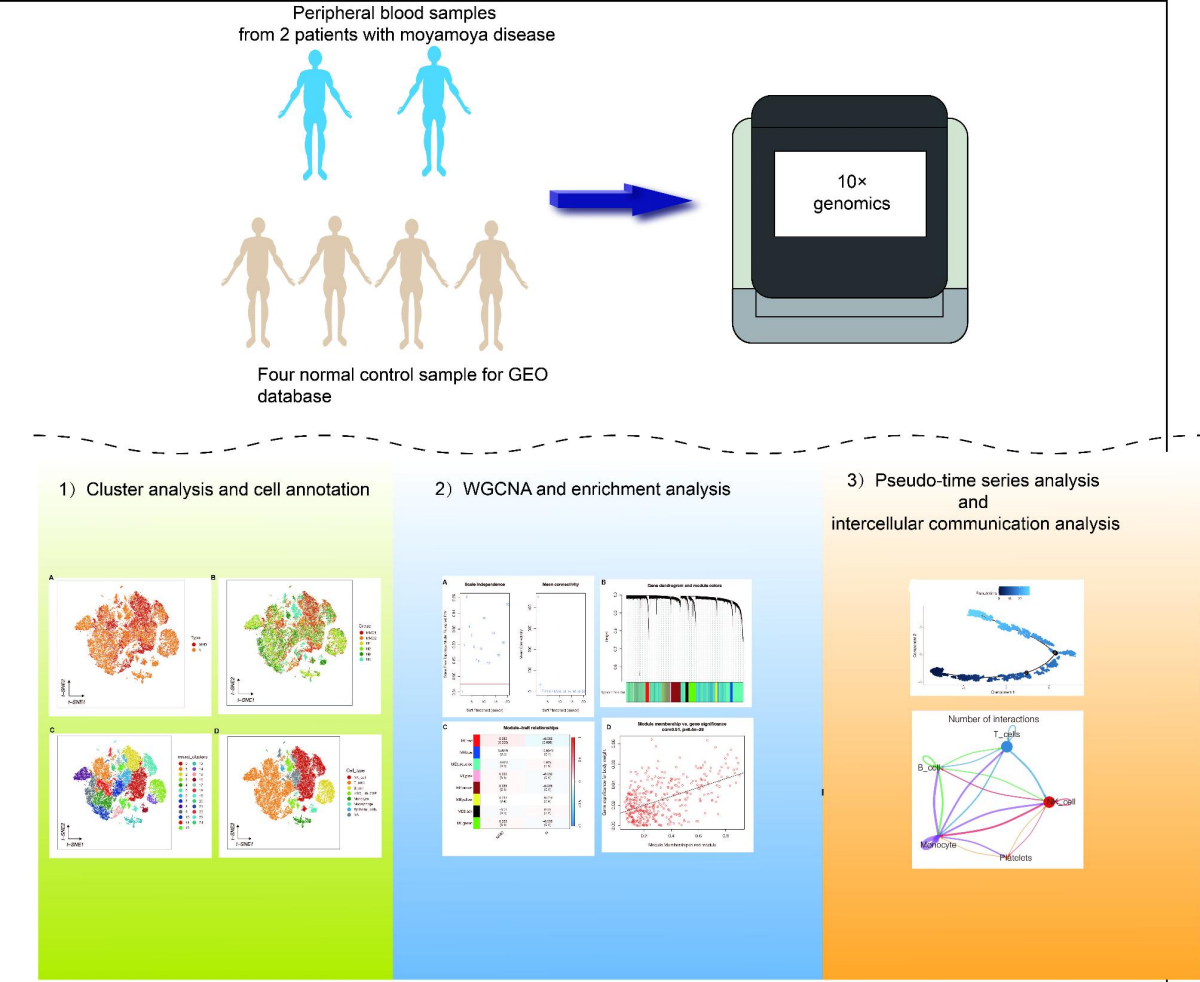
The flow chart

Figure 1 showed our workflow.

### Single cell sequencing data analysis

**Fig. 2:**
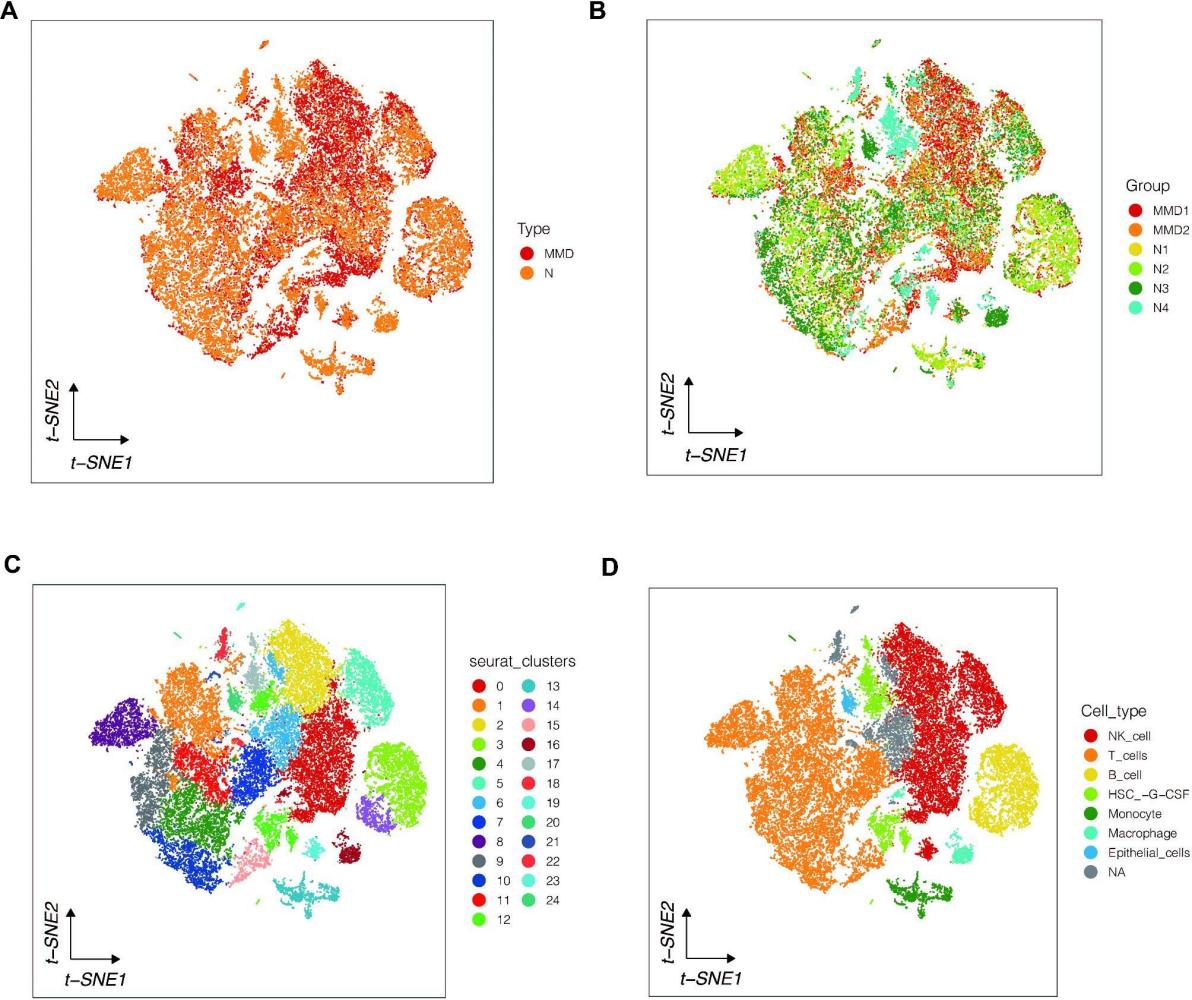
Single cell sequencing data analysis. (**A**) Cells obtained from Moyamoya disease (MMD) and normal controls (N). (**B**) Batch effect testing. (**C**) All cells were clustered into 25 clusters. (**D**) Cell annotation

**Fig. 3:**
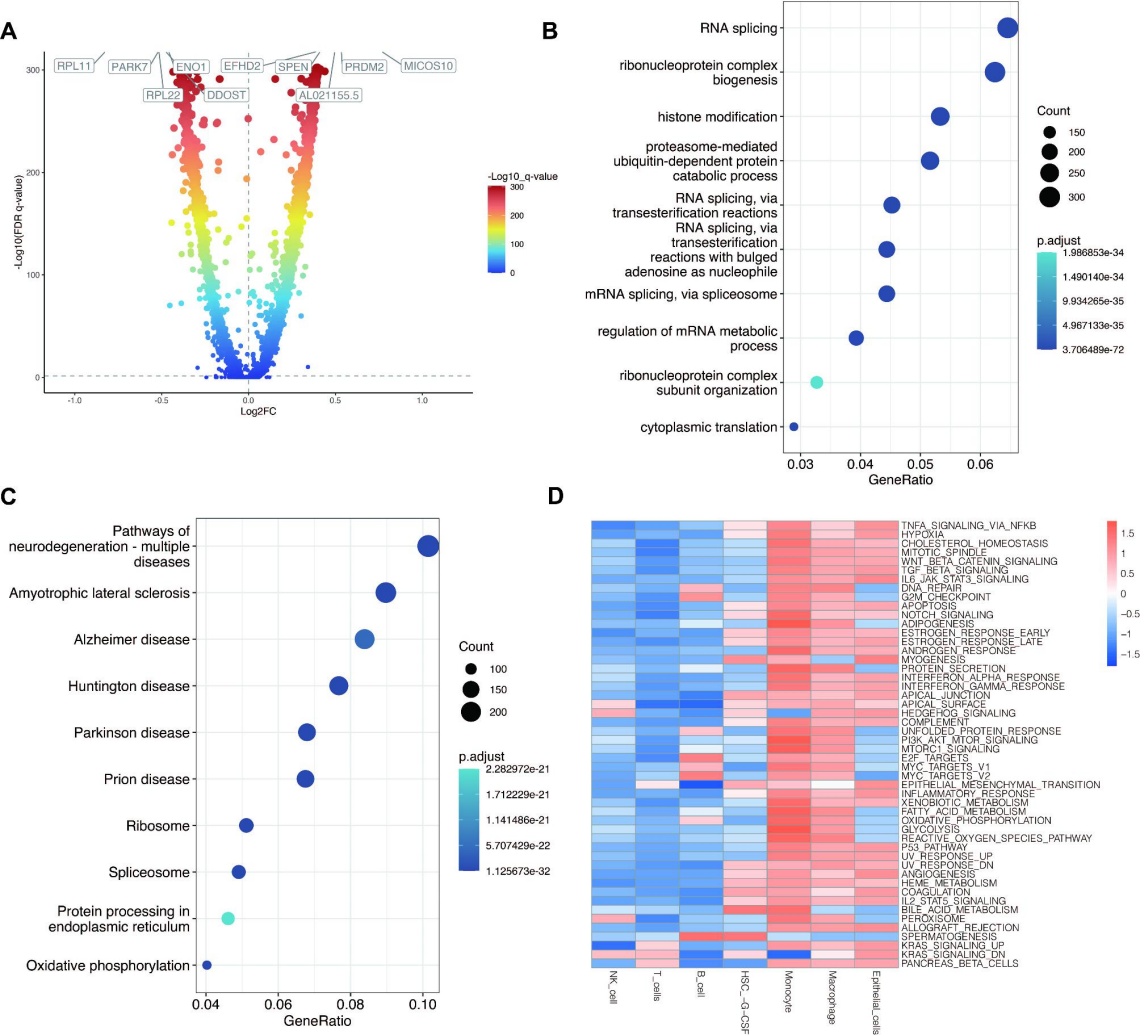
Differentially expressed genes and their enrichment analysis. (**A**) Analysis of differentially expressed genes between moyamoya disease and normal controls. (**B**) GO enrichment analysis of differentially expressed genes. (**C**) KEGG enrichment analysis of differentially expressed genes. (**D**) Pathway enrichment of different immune cells

In this study, two samples of Moyamoya disease and four normal samples were analyzed first. Through quality control, a total of 15,459 cells of Moyamoya disease and 20,079 normal control cells were obtained, as shown in Fig. 2A. Red represents Moyamoya disease samples and yellow represents normal samples. As shown in Fig. 2B, no significant batch effect was observed between the two samples of Moyamoya disease. Similarly, no significant batch effect was observed among the four samples of the normal control, while there was heterogeneity between the moyamoya disease and normal samples. Through dimensionality reduction and cluster analysis, all cells were coclustered into 25 clusters (Fig. 2C). A total of 7 cell types have been annotated, as shown in Fig. 2D: NK cells, T cells, B cells, HSC-G-CSF cells, Monocyte cells, Macrophage cells, Epithelial cells. In order to further explore the pathway activated in moyamoya disease, we first obtained significantly up-regulated genes in moyamoya disease by differential expressed gene analysis. The first five up-regulated genes were RPL11,PARK7, ENO1, RPL22 and DDOST. The five genes that were significantly down-regulated were EFHD2, SPEN, AL02AA55.5, PRDM2 and MICOS10 (Fig. 3A). In Fig. 3B-C, GO and KEGG enrichment analysis showed that functional pathways associated with moyamoya disease include RNA splicing, ribonucleoprotein complex biogenesis, and pathways of neurodegeneration multiple diseases, amyotrophic lateral sclerosis, etc. Then, we screened all moyamoya cells and explored the functional pathways. As shown in Fig. 3D, we found that most of the pathways were highly activated in Monocyte cells, Macrophage cells and Epithelial cells. However, the activation degree of NK cells, T cells and B cells was lower.

### Weighted co-expression Network Analysis (WGCNA), functional enrichment and search for key genes

**Fig. 4:**
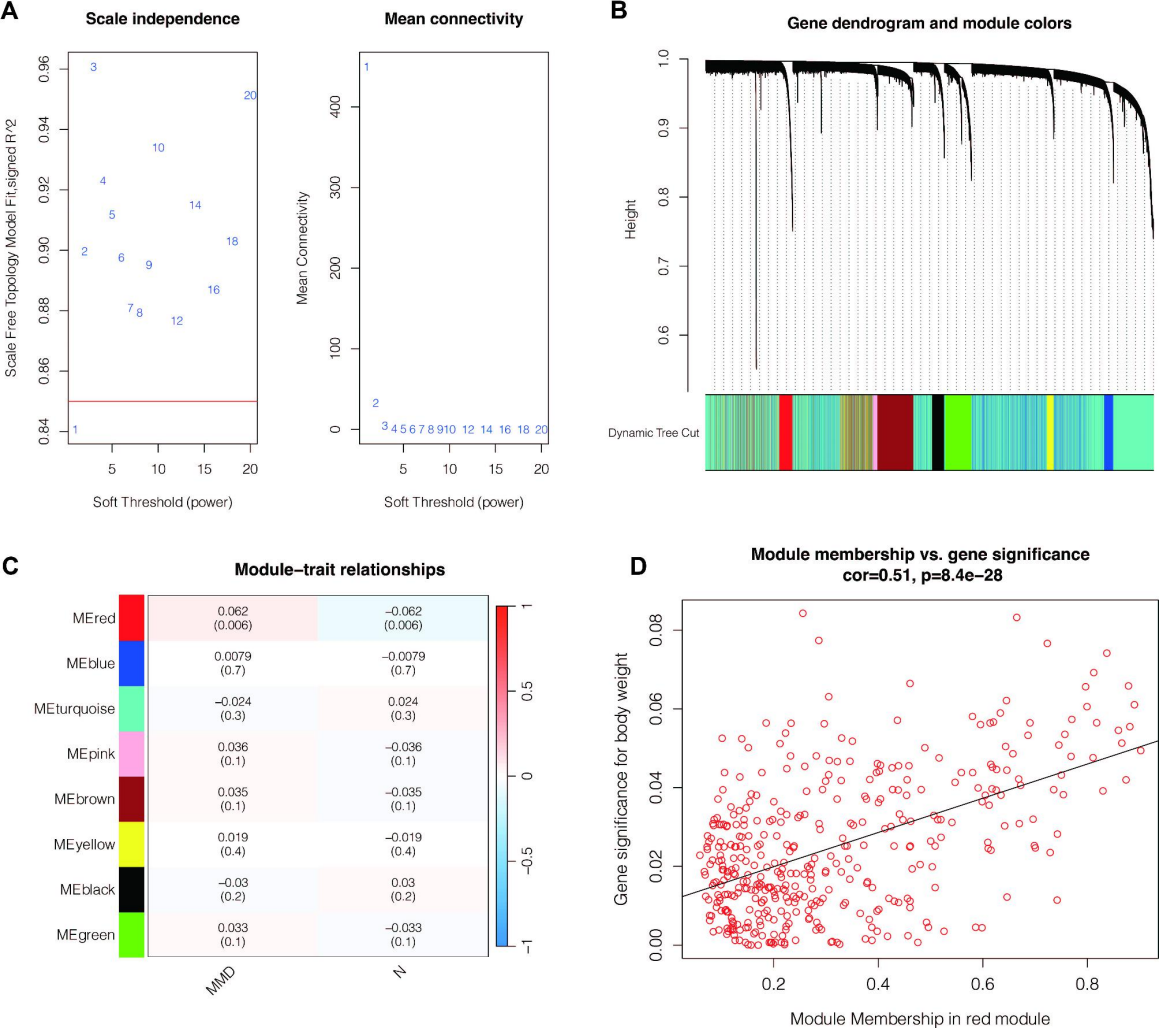
Weighted Coexpression network analysis (WGCNA). (**A**) Determination of optimal soft thresholds. (**B-C**) Cluster integration of all genes. The red module is considered the critical module. (**D**) Correlation of red modules

**Fig. 5:**
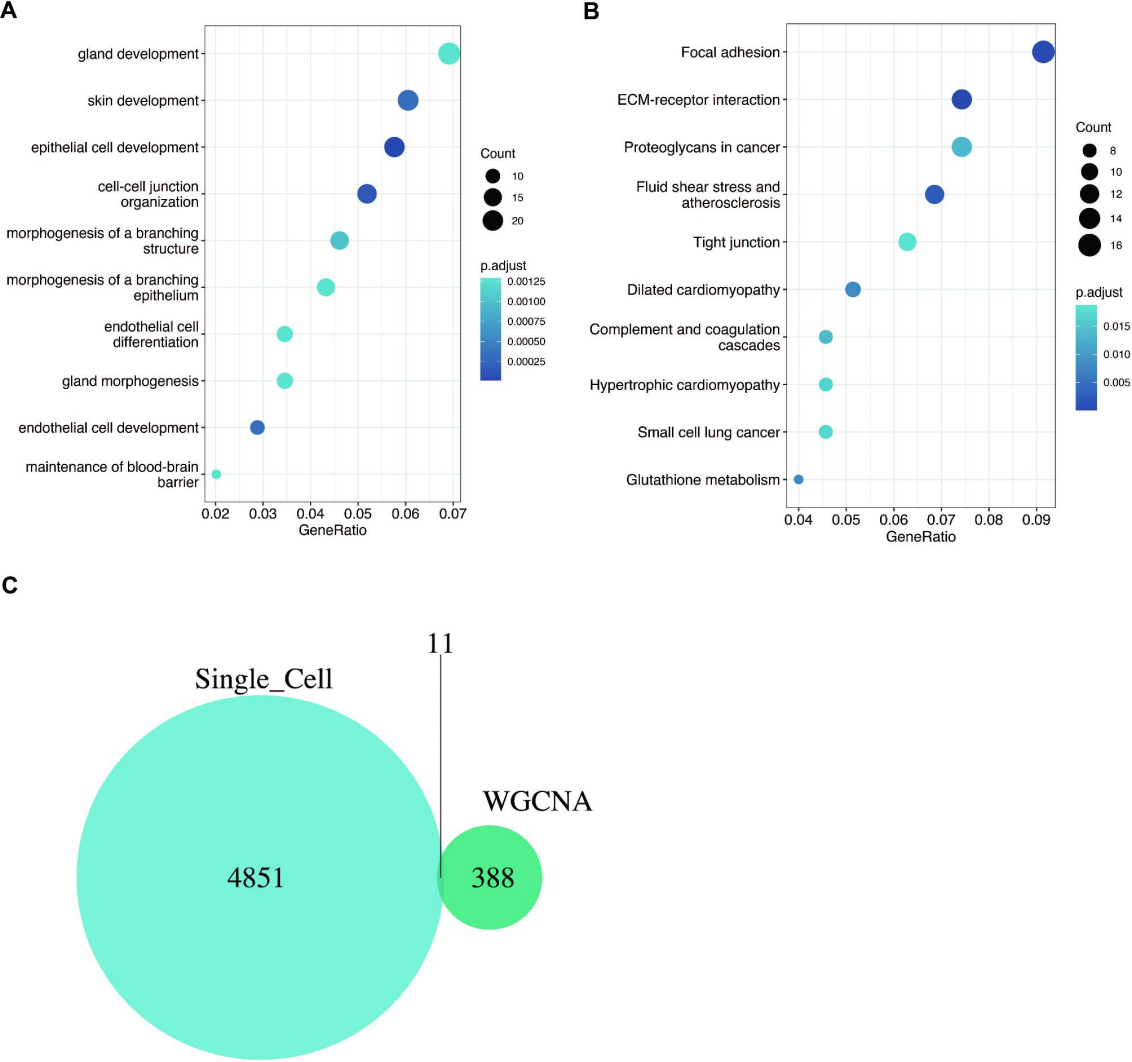
Enrichment analysis of red module genes obtained from WGCNA. (**A**) GO enrichment analysis. (**B**) KEGG enrichment analysis. (**C**) The intersection of red module genes of WGCNA and differentially expressed genes obtained by single-cell sequencing analysis resulted in 11 genes

Then we used the “sample” function to randomly select 2000 samples of Moyamoya disease cells and performed WGCNA analysis. As shown in Fig. 4A, the optimal soft threshold was 2, and the data conform to the power law distribution. As shown in Fig. 4B-C, genes were aggregated into 8 non-gray modules, among which the red module was most closely related to the occurrence of moyamoya disease. As shown in Fig. 4D, module membership of genes in the red module was positively correlated with gene significance for body weight (cor = 0.51, p < 0.001). We then investigated the function of these red module genes, as shown in Fig. 5A-B. These genes are associated with gland development, epithelial cell development, cell-cell junction organization, focal adhesion, and ECM-receptor interaction. Then, we intersected the differential genes obtained from single-cell analysis with the red module genes, and a total of 11 genes were obtained. They were respectively PTP4A1, SPINT2, CSTB, PLA2G16, GPX1, HN1, LGALS3BP, IFI6, NDRG1, GOLGA2, LGALS3(Fig. 5C).

### Pseudo-time series analysis

**Fig. 6:**
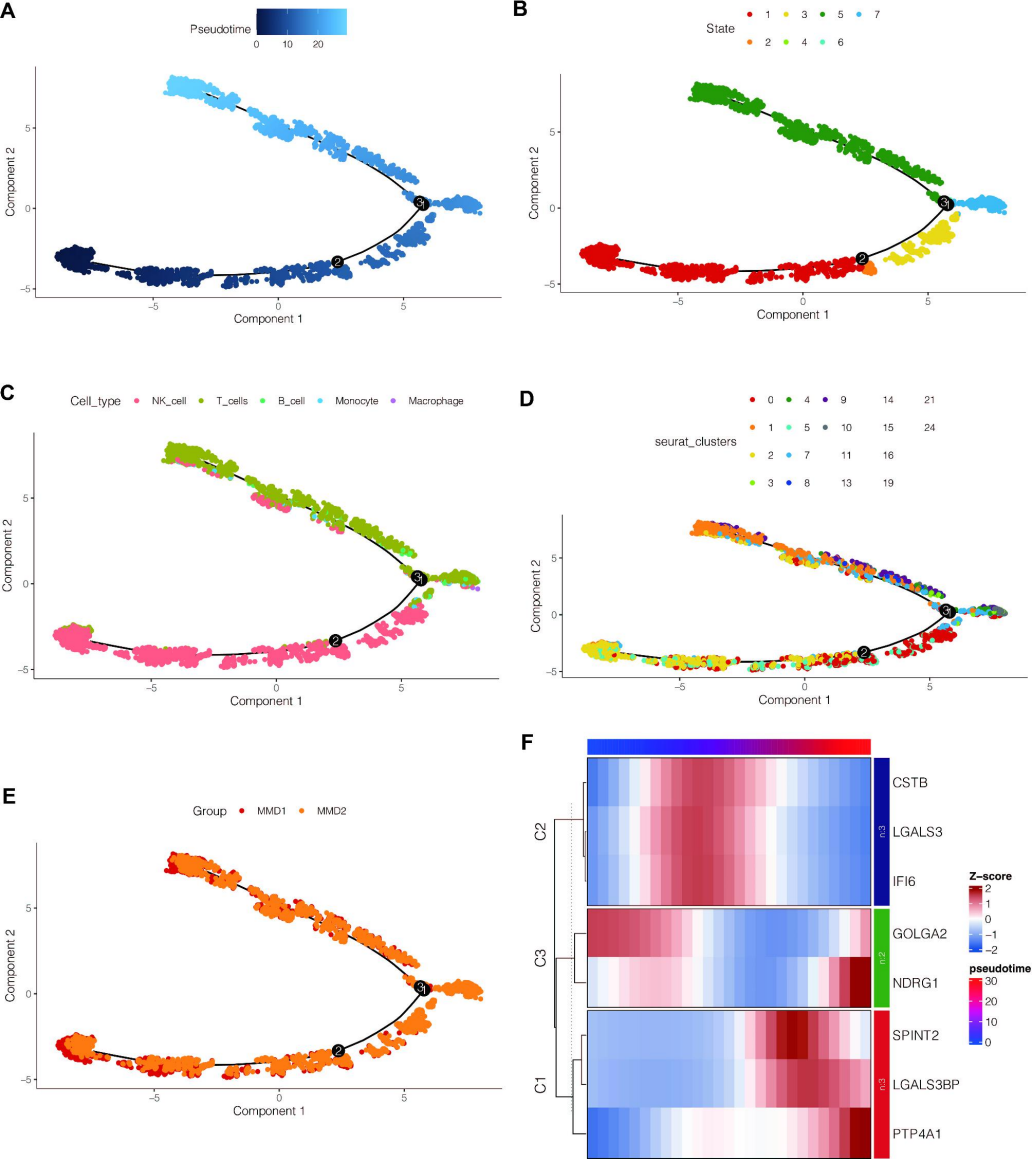
Pseudo-time series analysis. (**A**) Darker blue indicates early cell differentiation, from dark blue to light blue. (**B**) The cells have a total of seven differentiated states. (**C, D**) NK cells differentiated early, corresponding to state 1, corresponding to two major clusters (**E**) The differentiation states of the two Moyamoya disease samples were consistent. (**F**) The expression of 8 of the 11 key genes was changed. Among them, the expressions of CSTB, LGALS3 and IFI6 genes were firstly increased and then decreased, the expressions of GOLGA2 and NDRG1 were firstly decreased and then increased, and the expressions of SPINT2, LGALS3BP and PTP4A1 were gradually increased

Subsequently, immune cells were extracted from samples of Moyamoya disease for pseudo-time series analysis. As shown in Fig. 6A, darker blue means that cells differentiate earlier, from dark blue to light blue. These cells had a total of seven differentiation states (Fig. 6B). NK cells differentiated early, corresponding to differentiation state 1, and corresponding to two main clusters (Fig. 6C and D). Moreover, we found that the differentiation status of the two samples of Moyamoya disease was consistent (Fig. 6E). As shown in Fig. 6F, we found that the expression of 8 of the above 11 important genes was changed during the differentiation of immune cells. Among them, the expression of genes CSTB, LGALS3 and IFI6 increased first and then decreased, while the expression of GOLGA2 and NDRG1 decreased first and then increased, while the expression of SPINT2, LGALS3BP and PTP4A1 increased gradually.

### Cell interaction analysis

**Fig. 7:**
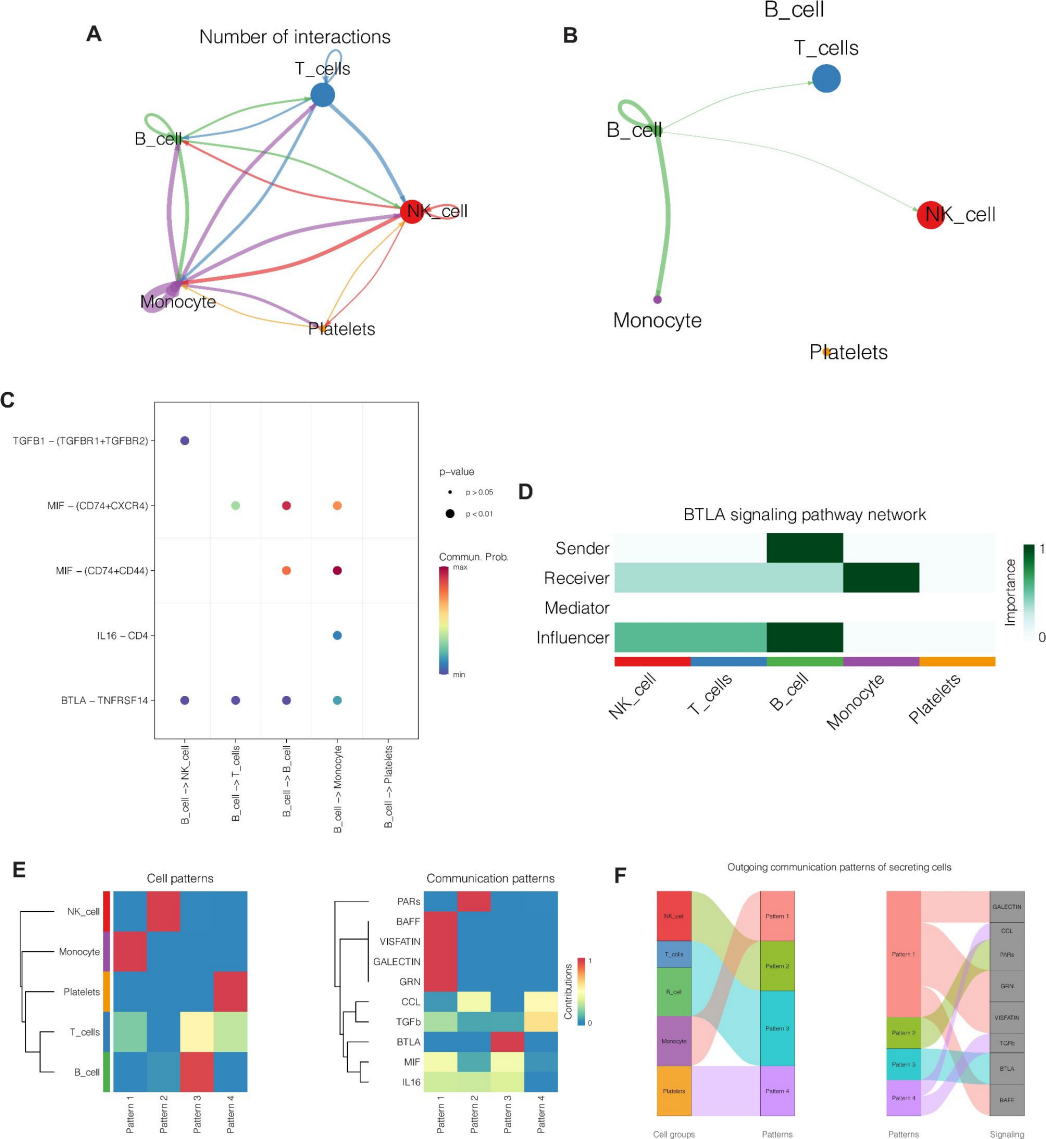
Cell interaction analysis. (**A**) The correlation between the five cell types. (**B**) B cells are mainly associated with T cells, NK cells and Monocyte cells, of which Monocyte is the most closely associated. (**C**) The BTLA-TNFRSF14 pathway is involved in the connection between B cells and other cells, suggesting that this pathway may play an important role. (**D**) In the BTLA pathway, B cells mainly send and affect signal transmission, while Monocyte cells mainly receive signals. (**E**) Cells are divided into parten1-4. Among them, B cells are mainly in parten3, and the main gene expressed in parten3 is BTLA. (**F**) Sankey diagram similarly shows that B cells correspond to parten3, and BTLA is mainly expressed in parten3

Cell interaction analysis was performed to further explore the connections between various cells in Moyamoya disease. Figure 7 A showed the correlation between the five cell types and to what extent. The thicker the line, the closer the connection. As shown in Fig. 7B, B cells were mainly associated with T cells, NK cells and Monocytes, of which Monocyte was the most closely associated. As shown in Fig. 7C, the BTLA-TNFRSF14 pathway was involved in the connection between B cells and other cells, suggesting that this pathway may play an important role. As shown in Fig. 7D, in the BTLA pathway, B cells mainly send and affect signal transmission, while Monocyte cells mainly receive signals. As shown in Fig. 7E, according to the degree of cell communication, cells were divided into four modes, distributed as parten1-4. Among them, B cells were mainly in parten3, and the main gene expressed in parten3 is BTLA. As shown in Fig. 7F, Sankey diagram similarly showed that B cells correspond to parten3, and BTLA was mainly expressed in parten3.

## Discussion

Stroke is one of the main causes of disability or death in middle-aged and elderly people, with high morbidity, mortality and disability rates [24]. Although most strokes are caused by high blood pressure, they can also be caused by an easily overlooked disease: Moyamoya disease [25]. Stroke caused by Moyamoya disease is associated with progressive narrowing of the intracranial internal carotid artery and its proximal branch [26]. Although vascular reconstructive surgery is capable of improving the progression of Moyamoya disease, identification of the disease at an early stage is critical in order to achieve the best results in patients [27]. However, the pathogenesis of moyamoya disease is still unclear at present, and multi-omics studies such as genome and transcriptome are urgently needed to reveal the potential mechanism of moyamoya disease formation, so as to provide reference data for the early diagnosis and treatment of Moyamoya disease.

For the first time, we explored the single-cell sequencing map of moyamoya disease peripheral blood using a variety of algorithms, including dimension reduction clustering, enrichment analysis, differential expression gene analysis, weighted coexpression network analysis, and intercellular communication analysis. Our results for the first time identified cellular heterogeneity in Moyamoya disease peripheral blood and identified potential key genes PTP4A1, SPINT2, CSTB, PLA2G16, GPX1, HN1, LGALS3BP, IFI6, NDRG1, GOLGA2, LGALS3. Moreover, the interaction between cells was clearly demonstrated, which provides a reference for us to understand the pathogenesis and the crosstalk between cells and pathways. This not only provided reference for us to understand the pathogenesis of moyamoya disease, but also provided us with a target pathway.

This study was the first single-cell sequencing analysis of Moyamoya disease. Compared with previous bulk RNA sequencing alone, our study was the first to cluster Moyamoya disease samples into different cell types through single-cell sequencing analysis, greatly reducing the heterogeneity of sequencing data, and studying the interaction mode between different cells. The BTLA-TNFRSF14 pathway identified by us may be a potential target for the treatment of Moyamoya disease. However, there are limitations to our study. First of all, due to the rarity of Moyamoya disease, we can obtain a small number of samples. Second, our normal controls came from a public database. We will expand the sample size and conduct further in-depth analysis in the future.

## Conclusion

This study was the first single-cell sequencing analysis of Moyamoya disease. We explored the single-cell sequencing map of moyamoya disease peripheral blood using a variety of algorithms, including dimension reduction clustering, enrichment analysis, differential expression gene analysis, weighted coexpression network analysis, and intercellular communication analysis. Our results for the first time identified cellular heterogeneity in Moyamoya disease peripheral blood and identified potential key genes.

## Data Availability

All the data can be found in the article. The normal sample was downloaded from GEO, accession number GSE168732(https://www.ncbi.nlm.nih.gov/geo/query/acc.cgi?acc=GSE168732).
